# Sulfhydration-associated phosphodiesterase 5A dimerization mediates vasorelaxant effect of hydrogen sulfide

**DOI:** 10.18632/oncotarget.16649

**Published:** 2017-03-29

**Authors:** Yan Sun, Yaqian Huang, Wen Yu, Siyao Chen, Qiuyu Yao, Chunyu Zhang, Dingfang Bu, Chaoshu Tang, Junbao Du, Hongfang Jin

**Affiliations:** ^1^ Department of Pediatrics, Peking University First Hospital, Beijing, 100034, China; ^2^ Department of Cardiac Surgery, Guangdong General Hospital, Guangzhou, 510000, China; ^3^ Centre Laboratory of Peking University First Hospital, Beijing, 100034, China; ^4^ Department of Physiology and Pathophysiology, Peking University Health Science Center, Beijing, 100034, China; ^5^ Key Laboratory of Cardiovascular Sciences, Ministry of Education, Beijing, 100034, China

**Keywords:** hydrogen sulfide, sGC/PDE/cGMP/PKG, dimer, sulfhydrylation

## Abstract

The study was designed to examine if the vasorelaxant effect of hydrogen sulfide was mediated by sulfhydration-associated phosphodiesterase (PDE) 5A dimerization. The thoracic aorta of rat was separated and the vasorelaxant effects were examined with *in vitro* vascular perfusion experiments. The dimerization and sulfhydration of PDE 5A and soluble guanylatecyclase (sGC) were measured. PDE 5A and protein kinase G (PKG) activities were tested. Intracellular cGMP content was detected by enzyme-linked immunosorbent assay (ELISA). The results showed that NaHS relaxed isolated rat vessel rings at an EC50 of (1.79 ± 0.31)×10^−5^mol/L, associated with significantly increased PKG activity and cGMP content in vascular tissues. Sulfhydration of sGC β1 was increased, while the levels of sGC αβ1 dimers were apparently decreased after incubation with NaHS in vascular tissues. Moreover, PDE 5A homodimers were markedly decreased, and accordingly the PDE 5A activity demonstrated by the content of 5′-GMP was significantly decreased after incubation with NaHS or GYY4137. Mechanistically, both NaHS and GYY4137 significantly enhanced the PDE 5A sulfhydration in vascular tissues. DTT partially abolished the effects of NaHS on PDE 5A activity, cGMP content and vasorelaxation. Therefore, the present study for the first time suggested that H_2_S exerted vasorelaxant effect probably via sulfhydration-associated PDE 5A dimerization.

## INTRODUCTION

H_2_S, previously known as a waste gas, has recently been established by several studies as an important gasotransmitter endogenously generated and involved in the regulation of physiological and pathophysiologic processes. It was revealed that endogenous H_2_S could reduce blood pressure, relax blood vessels, and inhibit the proliferation of smooth muscle cells, demonstrating important roles in cardiovascular regulation [1–17]. Previous study revealed the vasorelaxant effects of H_2_S by targeting potassium channel and L-calcium channel pathway in vascular smooth muscle cell membrane [18–24]. Meanwhile, more and more studies showed that H_2_S could also target other pathways to relax blood vessels [25–27]. The soluble guanylatecyclase (sGC)/cyclic nucleotide phosphodiesterase (PDE)/cyclic guanosine monophosphate (cGMP)/protein kinase G (PKG) pathway is one of the signal transduction pathways relating with vascular relaxation. Intracellular cGMP concentration was modulated by the balance between cGMP synthesis and degradation. GC is a key enzyme catalyzing cGMP synthesis. There are two types of GC, membrane-bound GC and sGC, with the latter playing an important role in regulating vasodilation. The sGC protein is formed by α and β subunits which can catalyze cGMP generation from guanosine-5′-triphosphate (GTP) upon activation. 5′GMP is generated from the degradation of cGMP by PDEs, which is one of the important negative regulatory factors in the cGMP signal pathway.

Recently, Gao, et al. discovered another mechanism for sGC activation, namely sulfhydryl-dependent dimerization formation. In this mechanism, heterodimers are formed by disulfide bonds of sGC protein α and β subunits, activating sGC and its downstream pathway, and thus resulting in vasodilatory effects. Similar to sGC, the other key regulatory molecules in sGC/PDE/cGMP/PKG pathway, such as PDE are also constituted of two subunits, regulating its activity by the disulfide bond-dependent heterodimer formation [28]. Previous studies showed that H_2_S could regulate protein function via modifying the thiol group of protein (-SSH) [29, 30]. Zhao, et al. found that H_2_S could affect the formation of disulfide bond on androgen receptor (AR) by its S-sulfhydration [31]. A previous study demonstrated that exposure of cells to physiological amounts of endogenous H_2_S increased intracellular cGMP levels [24]. They showed that the eNOS/cGMP/PKG axis was emerging as an important component of H_2_S signaling and biological activity [32]. Sofia-Iris, et al. found that NaHS could activate PKG both in cGMP-dependent and independent manners [32]. However, whether H_2_S exerted its vasorelaxant effects through inhibiting the disulfide bond-dependent heterodimer formation of sGC and/or PDE is yet to be explored. The present study was, therefore, designed to examine if H_2_S exerts vasorelaxant effect by PDE dimerization in association with its sulfhydration.

## RESULTS

### The vasorelaxant effect of NaHS

**Figure 1:**
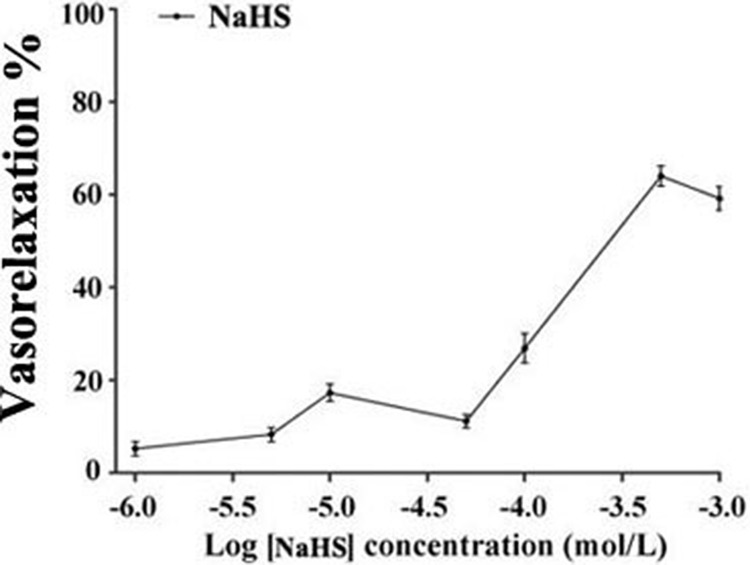
The vasorelaxant effect of NaHS in rat thoracic aorta rings (*n* = 8)

It was found that NaHS relaxed isolated rat thoracic aorta rings at an EC50 of (1.79 ± 0.31)×10^−5^mol/L. The maximum diastolic effect of NaHS was (60.49 ± 1.48)% at the concentration of 500 μmol/L. Whereas, the vasorelaxant effect of NaHS was (55.71 ± 1.65)% at a concentration of 1000 μmol/L (*n* = 8, Figure 1).

### NaHS enhanced PKG activity

**Figure 2:**
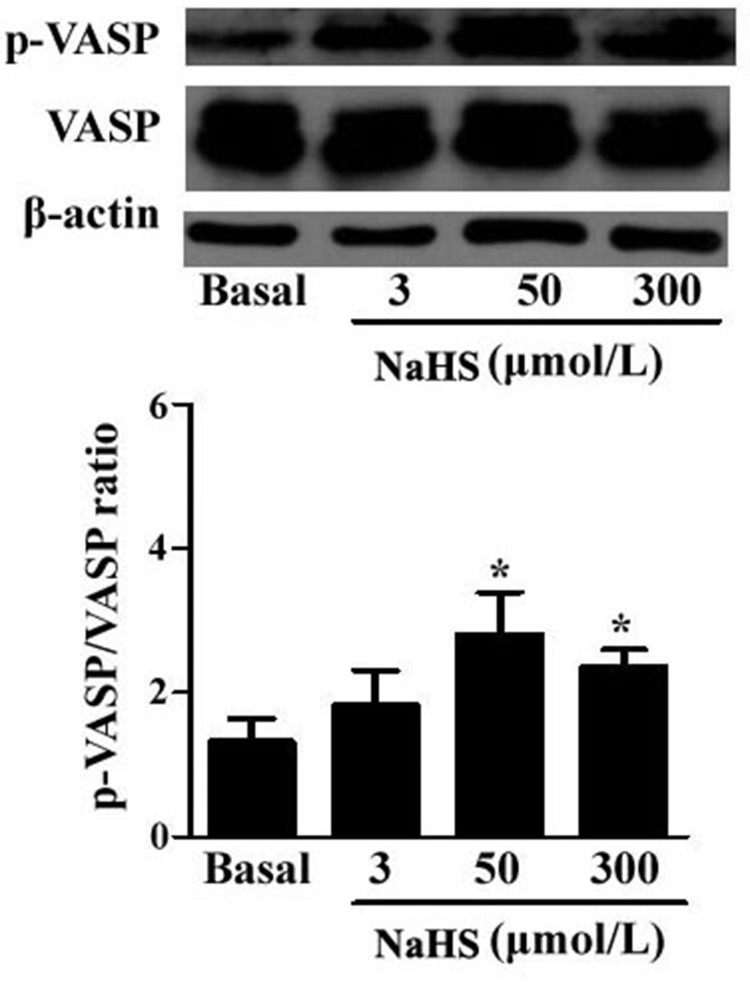
NaHS enhanced the phosphorylation level of VASP in vascular tissues (*p < 0.05 as compared with the basal group, *n* = 8)

PKG is a key molecule in the sGC/cGMP signaling pathway. We determined the effects of NaHS on PKG activity in the vascular tissues by detecting the phosphorylation level of vasodilator-stimulated phosphoprotein (VASP) using Western blotting. The results demonstrated that compared with the basal line, after incubation with NaHS at the concentration of 50 and 300 μmol/L for 15 min, the p-VASP/VASP ratio was significantly increased by 112.9% and 78.8%, respectively (*P* < 0.05, *n* = 8, Figure 2), indicating a significantly increased PKG activity in vascular tissues.

### NaHS increased cGMP level in aortic rings

To further explore the mechanism for vasodilation induced by NaHS, we examined cGMP level in vascular tissues. The results showed that compared with the basal line, after incubation with NaHS at 50 and 300 μmol/L for 15 min, the cGMP content in vascular tissue was increased by 130.8% and 89.2%, respectively (*P* < 0.01, *n* = 8, Table 1), suggesting that NaHS increased cGMP content in aortic rings.

**Table 1:** NaHS increased cGMP level in aortic rings (n = 8, mean ± SEM)

NaHS (μmol/L)	cGMP content (pmol/mg)	*P*
Basal (0)	25.38 ± 2.46	
3	23.22 ± 3.50	0.51
50	58.57 ± 5.71**	0.001
300	48.03 ± 3.72**	0.001

**P < 0.01, compared with the basal group, *n* = 8

### Both NaHS and GYY4137 reduced sGC αβ1 dimerization in vascular tissues

**Figure 3:**
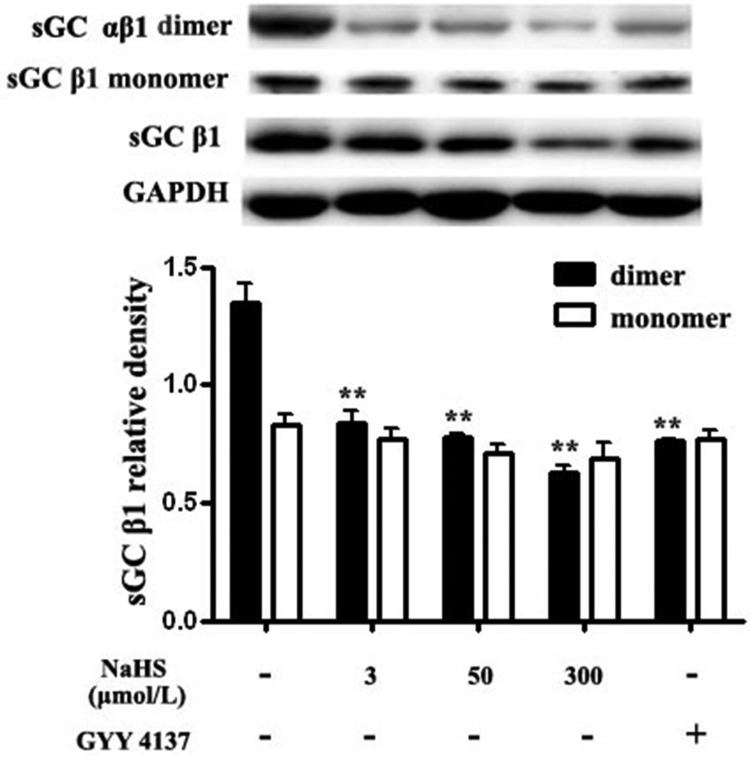
Both NaHS and GYY4137 reduced sGC αβ1 dimerization in vascular tissues **p < 0.01, compared with the basal group, *n* = 8.

The synthesis of cGMP is catalyzed by sGC which consists of an α (α1 or α2) and a β subunit (β1). The dimerization of α and β1 subunits of sGC is required for its catalytic activity. Therefore, we detected the level of sGC β1 monomer and sGC αβ1 dimer with antibody of sGC β1 by non-denaturing Western blotting method. We found that compared with the basal value, after incubation with NaHS at 3, 50 and 300 μmol/L for 15 min, the levels of sGC dimers in vascular tissues were significantly decreased by 37.9%, 42.4% and 53.3%, respectively (*P* < 0.05, *n* = 8, Figure 3). Compared with the basal value, the level of sGC αβ1 dimer after the incubation with GYY4137, at a concentration of 200 μmol/L in vascular tissues was significantly decreased by 43.6% (*P* < 0.01, *n* = 8, Figure 3). While, compared with the basal value, after incubation with NaHS at 3, 50 and 300 μmol/L or with GYY4137 at 200 μmol/L for 15 min, the levels of sGC β1 monomers in vascular tissues did not change (*P* > 0.05, *n* = 8, Figure 3).

### NaHS improved the sulfhydration of sGC β1 in vascular tissues

**Figure 4:**
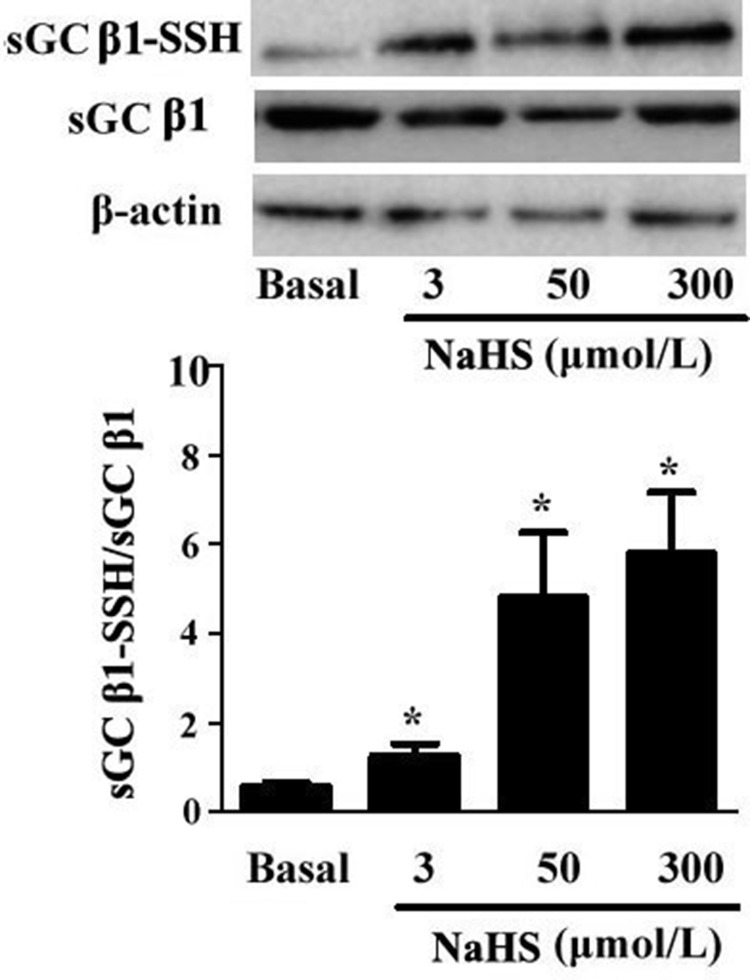
NaHS upregulated sulfhydryl modification of sGC β1 *p < 0.05, compared with the basal group, *n* = 8.

Most effects of H_2_S are mediated through post-translational modification of proteins by sulfhydration. Here, we found that the sulfhydryl modification of sGC β1 in vascular tissues was up-regulated with NaHS in a dose-dependent manner. As compared with the basal line, after incubation with NaHS at concentrations of 3, 50 and 300 μmol/L for 15 min, the sulfhydration in sGC β1 was increased by 117.2%, 727.5%, and 900%, respectively (*P* < 0.05, *n* = 8, Figure 4).

### Both NaHS and GYY4137 inhibited the formation of PDE 5A homodimers, suppressed PDE 5A activity and reduced cGMP degradation in vascular tissues

In addition to sGC-mediated synthesis, the content of cGMP was also dependent on degradation by PDE 5A. We tested the effect of NaHS on PDE 5A activity by detecting the content of cGMP degradation to 5′-GMP in a fixed frame of time. The data showed that compared with the basal line, 5′-GMP content in vascular tissues was decreased by 66.7% and 53.3% after the incubation with 50 and 300 μmol/L NaHS for 15 min, respectively (*P* < 0.05, *n* = 8, Table 2), indicating that NaHS inhibited PDE 5A activity and reduced cGMP degradation. To exclude the effect of sGC, we directly detected the activity of purified PDE 5A in a cell-free assay. In accordance with the above results, NaHS at the concentrations of 3, 50 and 300 μmol/L as well as GYY4137 significantly inhibited the activity of purified PDE 5A (*P* < 0.01, *n* = 8, Table 3).

**Table 2:** NaHS reduced PDE activity in aortic rings (n = 8, mean ± SEM)

NaHS (μmol/L)	5′- GMP (μmol/L)	*P*
Basal (0)	0.45 ± 0.08	
3	0.31 ± 0.09	0.27
50	0.15 ± 0.04 **	0.01
300	0.21 ± 0.06*	0.03

*P < 0.05, **P < 0.01, compared with the basal group, *n* = 8

**Table 3:** Both NaHS and GYY4137 reduced the activity of purified PDE 5A (n = 8, mean ± SEM)

group	5′- GMP (μmol/L)	*P*
Control	25.93 ± 1.62	
NaHS 3 μmol/L	15.96 ± 0.31^*^	0.004
NaHS 50 μmol/L	15.20 ± 0.63^*^	0.002
NaHS 300 μmol/L	14.80 ± 0.70^*^	0.001
NaHS 50 μmol/L +DTT	19.81 ± 0.77^##^	0.006
GYY4137 200 μmol/L	11.36 ± 0.39^*^	0.0003

DTT: 2 mmol/L; ^*^P < 0.01 *vs* control group; ^##^*P* < 0.01 *vs* NaHS 50 μmol/L group.

**Figure 5:**
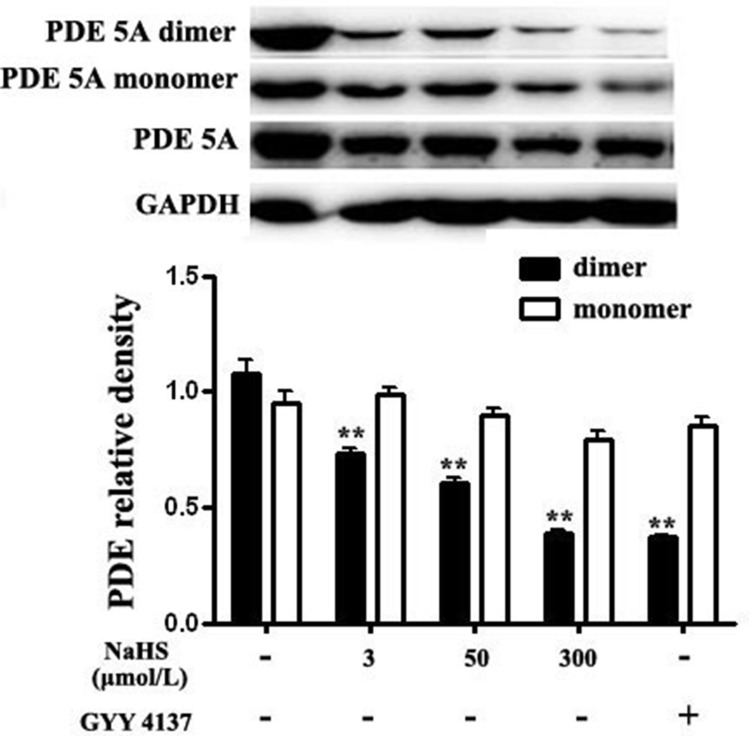
Both NaHS and GYY4137 inhibited the formation of PDE 5A homodimers in vascular tissues **p < 0.01, compared with the basal group, *n* = 8.

PDE 5A existed mainly as homodimers and the dimerization was closely associated with its degradative activity. We then examined the effect of NaHS on dimerization of PDE 5A. The results showed that compared with the basal line, after incubation with NaHS at concentrations of 3, 50 and 300 μmol/L for 15 min, the content of PDE 5A dimers was markedly decreased by 31.8%, 43.9% and 63.7%, respectively (*P* < 0.01, Figure 5). After incubation with 200 μmol/L GYY4137 for 15 min, the content of PDE 5A dimer was significantly decreased by 65.2% (*P* < 0.01, Figure 5). Whereas, compared with baseline, the incubation with different concentrations of NaHS or GYY4137 showed no effect on the expression of PDE 5A monomer (*P* > 0.05, Figure 5). These results indicated that NaHS and GYY4137 could inhibit the formation of PDE 5A homodimers, suppress PDE 5A activity and reduce cGMP degradation in vascular tissues.

### Both NaHS and GYY4137 improved the sulfhydryl modification of PDE 5A

**Figure 6:**
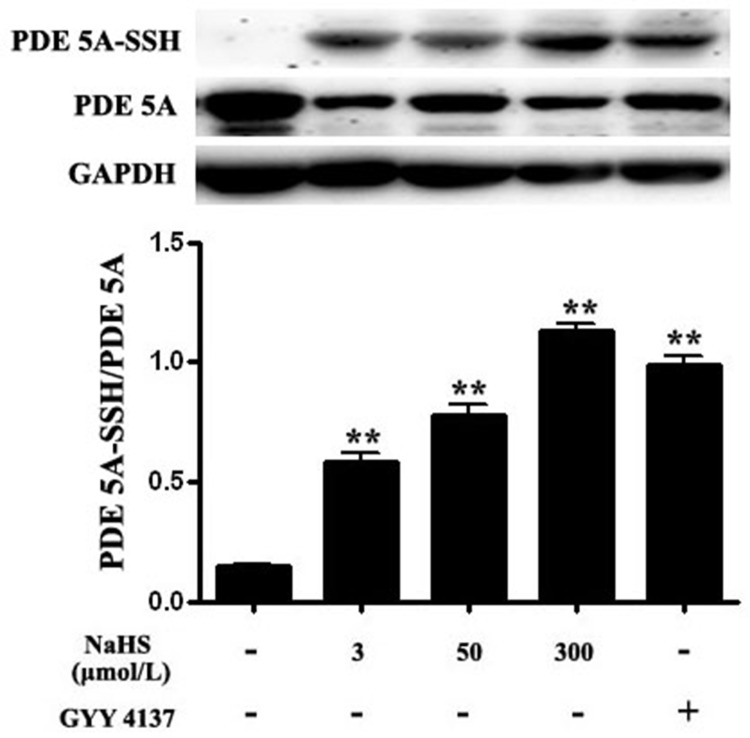
Both NaHS and GYY4137 upregulated sulfhydryl modification of PDE 5A **p < 0.01, compared with the basal group, *n* = 8.

The results showed that compared with the basal value, the ratio of sulfhydration of PDE 5A in vascular tissues was increased significantly by 301.1%, 438.5% and 379.9%, respectively, after incubation with NaHS at concentrations of 3, 50 and 300 μmol/L for 15 min (*P* < 0.01, *n* = 8, Figure 6). Similarly, compared with the basal value, the ratio of sulfhydration of PDE 5A in vascular tissues was increased significantly by 578.4% after the incubation with 200 μmol/L GYY4137 for 15 min (*P* < 0.01, *n* = 8, Figure 6). Moreover, DTT (2 mmol/L) treatment partly abolished the effects of 50 μmol/L NaHS on purified PDE 5A activity and vasorelaxation (Figure 8 and Table 3). These results suggested that NaHS sulfhydrated PDE 5A, then decreased PDE 5A activity and increased cGMP content, resulting in a vasorelaxation.

### Endogenous H_2_S inhibited the formation of sGC αβ1 dimers and PDE 5A homodimers but enhanced PDE 5A sulfhydration in aortic smooth muscle cells

**Figure 7:**
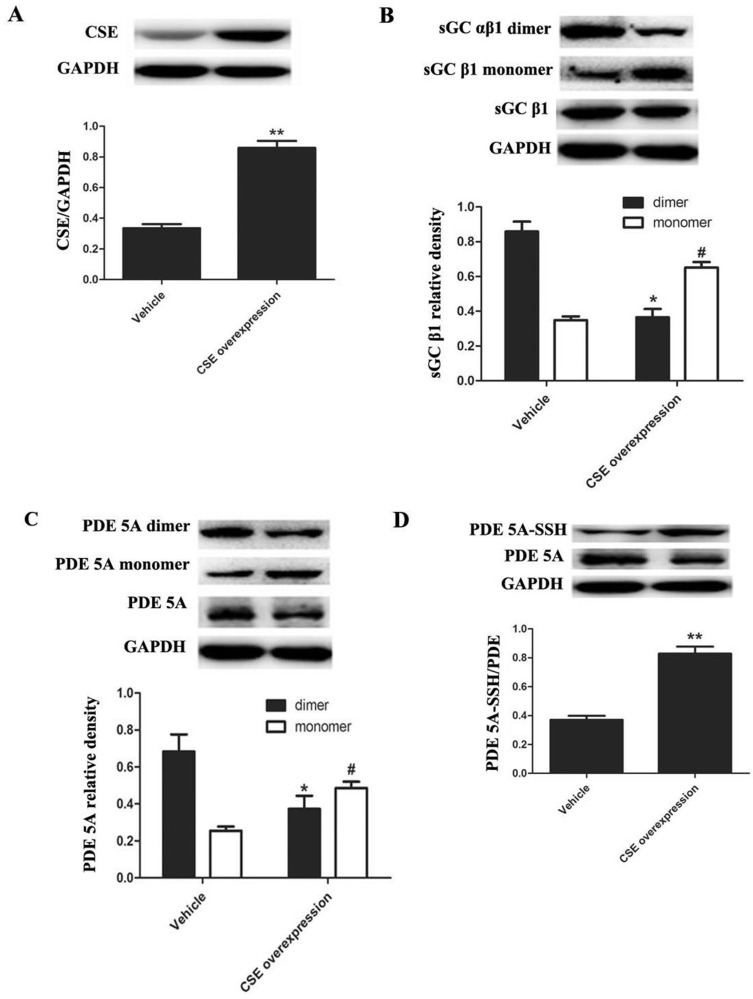
Endogenous H_2_S improved the sulfhydryl modification of PDE 5A and inhibited the dimerization of PDE 5A and sGC αβ1 in ASMCs (**A**) CSE overexpression increased CSE protein expression in ASMCs. *n* = 8, **p < 0.01, as compared with the vehicle group. (**B**) CSE overexpression decreased protein levels of sGC αβ1 dimers but increased monomer levels in ASMCs. *p < 0.05, as compared with the dimers of vehicle group; ^#^*p* < 0.05, as compared with the monomer of vehicle group. (**C**) CSE overexpression decreased protein levels of PDE 5A dimers but increased monomer levels in ASMCs. *p < 0.05, as compared with the dimers of vehicle group; ^#^*p* < 0.05, as compared with the monomer of vehicle group. (**D**) CSE overexpression enhanced the sulfhydration of PDE 5A in ASMCs. **p < 0.01, as compared with the vehicle group.

To detect the dimerization and sulfhydration of sGC β1 and PDE 5A in endogenous level, we overexpressed CSE, the key enzyme catalyzing endogenous H_2_S generation in rat aortic smooth muscle cells (ASMCs). Infection of ASMCs with CSE lentivirus markedly increased CSE protein expression compared with vehicle lentivirus infected ASMCs (*P* < 0.01, *n* = 8, Figure 7A). Compared with the vehicle group, the sGC αβ1 dimers were markedly decreased, while the sGC β1 monomer was significantly increased in ASMCs of CSE overexpression group (*P* < 0.05, *n* = 8, Figure 7B).

Similarly, compared with the vehicle group, the PDE 5A homodimers were markedly decreased, while the PDE 5A monomers were significantly increased in ASMCs overexpressing CSE (*P* < 0.05, Figure 7C). The sulfhydryl modification of PDE 5A in ASMCs overexpressing CSE was obviously increased compared with the vehicle group (*P* < 0.01, *n* = 8, Figure 7D). Thus, endogenous H_2_S could also enhance PDE 5A sulfhydration but inhibit PDE 5A and sGC αβ1 dimerization in ASMCs, which was consistent with that in vascular tissues studies.

### The vasorelaxant effect of NaHS was possibly in association with adenylyl cyclase pathway

**Figure 8:**
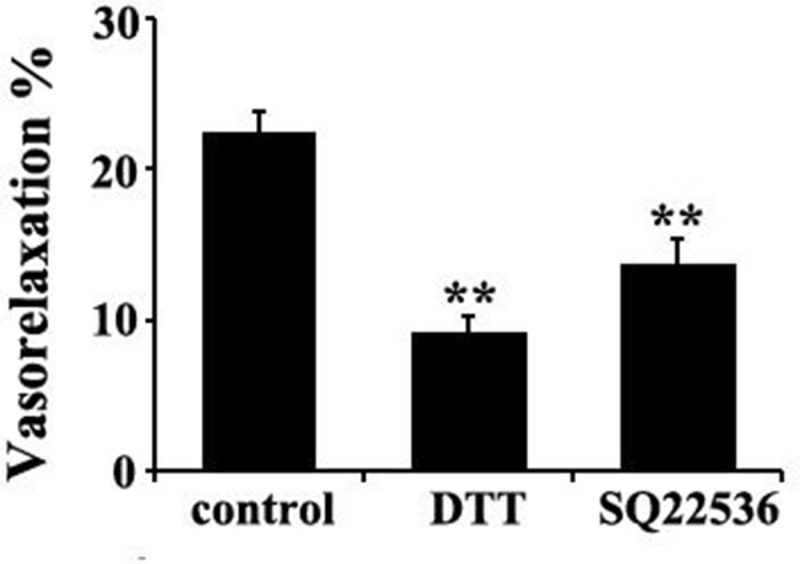
DTT or AC inhibitor SO22536 partly abolished the vasorelaxant effect of H_2_S in rat thoracic aorta rings **p < 0.01 compared with the control group, *n* = 8.

Considering H_2_S down-regulated adenylyl cyclase (AC)/cAMP pathway, we wondered if AC/cAMP pathway was involved in the mechanisms by which H_2_S induced vasorelaxation. The data showed that SQ22536, an AC inhibitor, at 100 μmol/L could significantly reduce the vasorelaxant effect of NaHS at a concentration of 50 μmol/L (*P* < 0.01, *n* = 8, Figure 8). Therefore, these results suggested that both cGMP and AC/cAMP pathway were involved in the vasorelaxant effect of NaHS.

## DISCUSSION

Recently, the role of H_2_S in cardiovascular regulation and its mechanisms has become an increasing hot issue in the field of cardiovascular research [33, 34]. It had been confirmed that K_ATP_ channel, L-type calcium channel and ion channels, etc. were involved in the vasorelaxant effect of H_2_S [35–37]. The sGC/PDE/cGMP/PKG pathway is one of the classical signaling transduction pathways of vascular relaxation. We wondered whether H_2_S exerted its vasorelaxant effects through inhibiting sulfhydryl-associated dimerization of sGC and/or PDE.

Previous studies reported that NaHS at the concentration of 3 μmol/L was the low limit value [38], while 50 μmol/L was the most commonly used physiological concentration [39], and 300 μmol/L was the high limit value in the physiological studies [40]. Therefore, we selected 3, 50 and 300 μmol/L as the three different concentrations of NaHS in the present study.

In this study, we showed that the vasorelaxant effect of H_2_S caused vasorelaxation in rat thoracic aortic rings *in vitro* in a dose-dependent manner, and its maximum diastolic effect on aortic rings was 55.71 ± 1.65% at the concentration of 500 μmol/L, whereas higher concentrations of NaHS failed to promote vasorelaxant effect (55.71 ± 1.65%).

To explore whether the PDE/cGMP/PKG pathway was involved in the vasorelaxant effect of H_2_S, in the light of the concentration range of the vasorelaxant effect of H_2_S and endogenous H_2_S level *in vivo* (55.01 ± 11.07 μmol/L), we mainly studied two concentrations of NaHS, 50 and 300 μmol/L, in the regulation of PDE/cGMP/PKG pathway in vascular tissues. Firstly, we examined cGMP content and PKG activity in vascular tissues. The results showed that compared with the baseline, both 50 and 300 μmol/L NaHS incubations enhanced cGMP content and PKG activity, indicating that H_2_S activated the cGMP/PKG pathway.

Next, we examined the possible mechanisms by which H_2_S increased cGMP content. Intracellular cGMP level was mainly maintained by the balance of its synthesis and degradation. The synthesis of cGMP from GTP is catalyzed by sGC. sGC is a heterodimer of an α subunit (α1 or α2) and a β subunit (β1). The dimerization of sGC αβ1 is essential for its catalytic activity. We found that NaHS and GYY4137 decreased sGC αβ1 dimerization in vascular tissues. The sGC αβ1 dimers were also reduced by endogenous H_2_S by CSE overexpression in ASMCs. These results indicated that H_2_S could inhibit sGC activity and cGMP synthesis. The observed rise in cGMP content in vascular tissues after incubation with NaHS dose not result from increased cGMP synthesis through sGC.

Intracellular cGMP concentration is modulated by the balance between cGMP synthesis and degradation. Thus, we explored the regulatory effect of H_2_S on the degradation of cGMP. It is known that PDE 5A is a key enzyme for cGMP degradation. Therefore, the activity of PDE 5A was detected in the study by measuring the rate of cGMP degradation to 5′ GMP in vascular tissues. The results showed that both NaHS and GYY4137 inhibited the rate of cGMP degradation to 5′ GMP, suggesting that H_2_S inhibited the activity of PDE 5A for cGMP degradation.

Up to now, the mechanisms by which H_2_S inhibited the activity of PDE 5A have been unclear. Recently, Gao, et al. found an interesting mechanism for the regulation of sGC and PDE 5A activity, namely sulfhydryl-dependent dimerization formation. Heterodimers are formed by disulfide bonds of sGC protein α and β subunits, which activates sGC and its downstream pathway, causing the relaxation of blood vessels [28]. We found that NaHS at concentrations of 3, 50 and 300 μmol/L enhanced the sulfhydration of sGC β1, further proving that NaHS could inhibit sGC β1 activity. Similar to sGC, PDE 5A also has two subunits, regulating its activity by the mechanism of disulfide bond-dependent heterodimer formation, which represented sGC and PDE 5A activation. The previous study showed that H_2_S could increase protein function by modifying the thiol group of protein (-SSH) [29, 30]. Meanwhile, Zhao, et al. found that H_2_S could improve the formation of disulfide bonds on androgen receptor (AR) by its S-sulfhydration [31]. Therefore, we further examined if H_2_S could inhibit PDE 5A dimerization in association with an enhanced sulfhydration. We interestingly found that NaHS, GYY4137 or endogenous H_2_S inhibited the PDE 5A dimerization, implying that H_2_S could inhibit PDE 5A dimerization and activity. Meanwhile, the sulfhydration of PDE 5A in vascular tissues was enhanced by NaHS or by GYY4137. The sulfhydryl modification of PDE 5A in vascular tissues was increased significantly after the incubation with NaHS at concentration of 3 μmol/L, 50 μmol/L, 300 μmol/L or 200 μmol/L GYY4137 for 15 min. Moreover, endogenous H_2_S also enhanced the sulfhydration of PDE 5A in ASMCs. The sulfhydration inhibitor DTT partly abolished the effects of NaHS on purified PDE 5A activity and vasorelaxation. The above mentioned results implied that H_2_S could promote PDE sulfhydration, inhibit its dimerization, and further reduce PDE activity, increasing cGMP content in vascular tissues.

Moreover, on the basis of the data showing that H_2_S inhibited the PDE 5A activity to reduce the cGMP degradation and suppressed sGC β1 activity to decrease the cGMP synthesis, the net effect after balance likely resulted in an increased cGMP level in vascular tissues, exerting the vasorelaxant effect of H_2_S.

In addition to cGMP, AC/cAMP is also a powerful vasodilatory signal. Previous studies reported that H_2_S played a role in the regulation of AC/cAMP pathway. We found that SQ 22536, an inhibitor of AC, could significantly reduce the vasorelaxant effect of NaHS, indicating that the vasorelaxant effect of H_2_S is mediated by both AC/cAMP and cGMP signaling pathways.

The present study also had limitations. The exact residue of PDE 5A responsible for this sulfhydration, and the possible residue of sulfhydration of a protein have not been clarified. Further studies were needed to clarify the exact residue of PDE.

In summary, the present study suggested that H_2_S enhanced PDE 5A sulfhydration to inhibit its dimerization and reduced its activity, accordingly inhibiting cGMP degradation, thereby activating the cGMP/PKG pathway for vascular relaxation. The study elucidated that the mechanism by which H_2_S exerted the vasorelaxant effect. It also provided potential targets of drug treatment in cardiovascular diseases by H_2_S in the future.

## MATERIALS AND METHODS

### Animals

Male adult Wistar rats (240–260 g) were purchased from the Experimental Animal Center of Peking University Health Science Centre and housed under standard conditions (constant temperature 27°C, 12:12-h light-dark), provided with adequate tap water and pelleted food. This study was approved by the Animal Research Ethics Committee of Peking University First Hospital (permit no.: J201437).

### Reagents

NaHS and GYY4137 were purchased from Sigma, freshly prepared with physiological saline before use. Phenylephrine hydrochloride (PE), dithiothreitol (DTT), S-methylmethanathiosufonate (MMTS), and PDE5A antibody were all purchased from Sigma. sGCβ1 antibody was bought from Cayman Chemica Company. PDE activity assay kit was purchased from EnzoBiochem Company. Anti-VASP and anti-phosphorylated VASP antibodies were purchased from Cell Signaling Company, β-actin monoclonal antibody from Santa Cruz company, and cGMP content assay kit from New East Biosciences Company. Conventional chemical reagents were all analytically pure and were purchased from Beijing Chemical Reagent (Beijing, China).

### Preparation of isolated aortic rings and detection of vascular tension

Urethane (12%, 0.1 ml/kg) was used for intraperitoneal anesthesia. Thoracic aorta was then quickly removed by thoracotomy and placed in pre-cold Krebs buffer (4°C, pH 7.4, 118.3 mM NaCl, 4.7mM KCl, 2.5 mM CaCl_2_, 1.2 mM MgSO_4_, 1.2 mM KH_2_PO_4_, 25 mM NaHCO_3_, and 11.1 mM glucose) with 95% O_2_ and 5% CO_2_ as described previously [41, 42]. Perivascular fat and connective tissues were carefully stripped to prepare approximately 3–5-mm aortic rings which were then affixed to a force transducer (model no. MLT0420, ADInstruments). Two triangular brackets horizontally passed through each aortic ring, immersed in an organ bath (Panlab 8 Chamber Organ Bath System, PL3508B6/C-V, ADInstruments, Sydney-NSW, Australia). They were immersed in an organ bath with 8 ml of Krebs buffer at 37°C gassed with 95% O_2_–5% CO_2_, which was helpful to keep tissue alive. The real-time tension change data were collected with a Force Transducer connected to a Power Lab Data Acquisition System (AD Instruments). The aortic rings were initially stretched passively to a tension of 1.5 g and allowed to equilibrate for 1 h. Then PE (1 μmol/L) was used to precontract the aortic rings. The concentration-response curve of H_2_S on the vasorelaxant effect was evaluated by cumulative addition of different concentrations of H_2_S after administration with PE. To examine whether the vasorelaxant effect of H_2_S was associated with its sulfhydration, DTT, the sulfhydration inhibitor (2 mmol/L) was added 30 min before the PE challenge. To examine the involvement of cAMP signaling, the AC inhibitor SQ22536 (100 μmol/L, dissolved in DMSO) was added 30 min before the PE challenge. The dilation effect was expressed as percentage PE contraction.

### Measurement of sGC β1 and PDE 5A expressions in aortic rings by non-denaturing Western blotting

Rat aortic rings were immersed in 95% O_2_-5% CO_2_ Kreb's buffer followed by incubation with various concentrations of NaHS (3, 50 and 300 μmol/L) for 15 min. The treated aortic tissues were frozen in liquid nitrogen, and non-reducing lysis buffer (50 mmol/L Tris·HCl, pH 7.5; 5 mmol/L EGTA, 2 mmol/L EDTA, 100 mmol/L NaF, 100 mmol/L maleimide) was added. After lysis and homogenization, the mixture was centrifuged at 13,000 g for 15 min at 4°C. The protein concentrations of the supernatants were determined using a BCA protein assay kit with BSA standards (Pierce Chemical, Rockford, IL). Protein samples (20 μg/lane) in non-denaturing buffer (Applygen, Beijing, China) were separated using 12% sodium dodecyl sulfate polyacrylamide gel electrophoresis (SDS-PAGE), and transferred to nitrocellulose membranes. The membranes were then blocked with TBS-Tween (TBST, 10 mM Tris·HCl, pH 8.0, 0.15 mM NaCl, and 0.05% Tween 20) containing 5% skim milk, and incubated with rabbit anti-sGCβ1 antibody (1:3,000 dilution), rabbit anti-PDE5A (1:1,000 dilution), and mouse anti-β-actin (1:3,000 dilution) overnight at 4°C, respectively. Afterwards, the blot was washed four times, each time 10 min, followed by addition of horseradish peroxidase-linked secondary antibodies for 60 min (Sigma-Aldrich, St. Louis, MO), and washed four times (×10 min). The bands were visualized using Super Signal West Pico Chemiluminescent Substrate Kit (Thermo Fisher Scientific). Band densities were detected by Kodak Image Station 4000 MM Digital Imaging System and analyzed by Alpha EaseFC software (AlphaInnotech, San Leandro, CA). The expression of β-actin was used as an internal control to normalize the expressions of target proteins. The percentage of relative density was calculated as the ratio of target protein expression to β-actin expression.

### Measurement of sulfhydryl groups in sGC β1 and PDE 5A by Western blotting

Rat aortic rings were incubated with different concentrations of NaHS (3, 50 and 300 μmol/L) for 15 min, respectively. Thoracic aortic rings were homogenized and extracted in ice-cold protein lysis buffer, and cell lysates were incubated with EZ-linkTMPEO-iodoacetyl biotin (10 mg/ml, Pierce) for 12 h at 4°C and then incubated with 20 μl of Ultra LinkTM Immobilized NeutravidinTM (Pierce) for 4 h on a roller system at 4°C. The beads were washed three times with 1 ml of ice-cold PBS. The mixtures were centrifuged at 5,000 g for 10 min at 4°C. The supernatants were removed, and 50 μl of lysis buffer added. The measurement of sulfhydryl groups in the sGC and PDE protein was analyzed by Western blotting and probed with rabbit anti-sGC and PDE antibody (Cell Signaling Technology).

### Detection of cGMP concentration

Aortic rings were reserved in liquid nitrogen immediately after incubation with saline or different concentrations of NaHS (3, 50 and 300 μmol/L) for 15 min, according to manufacture’ guidelines using cGMP kit (New East Biosciences, Malvern, PA). Lysis buffer with 0.1 M HCl was used to lyse the tissue, and after homogenization, centrifugation at 4°C (13, 000 g, 15 min) was used to collect the supernatant, which was then subject to detection of protein and cGMP concentration according to the manufacturer's instructions, using a commercial kit. The resultant absorbance was determined at 450 nm with a microplate reader (Bio-Rad Laboratories, Hercules, CA). A standard curve was prepared by plotting the absorbance value for each standard vs. its concentration, from which the cGMP concentration of each sample was determined.

### Detection of PDE activity

Aortic rings were rapidly frozen in liquid nitrogen after incubation with NaHS for 15 min and IBMX (100 μmol/L) for 30 min. The tissue was lysed with lysis buffer, and after homogenization and centrifugation at 13,000 g (4°C, 15 min), the supernatant was collected and used to detect PDE activity with a PDE activity assay kit (EnzoBiochem). Turpentine was used to remove phosphate anions from the sample prior to analysis. To detect the activity of purified PDE 5A, the PDE 5A from bovine brain was incubated with different concentrations of NaHS (3, 50 and 300 μmol/L), DTT (2 mmol/L) plus NaHS (50 μmol/L), or GYY4137 (200 μmol/L) for 1 h at room temperature. Thereafter, the cGMP substrate, 5′-nucleotidase, tissue samples or purified PDE 5A, or 5′-GMP standards were added, according to the kit requirements, then incubated with shaking for 30 min at room temperature. Biomol Green reagent was added after 60 min, and the resultant absorbance was measured at 620 nm with a microplate reader after incubation for 30 min. A standard curve was prepared by plotting the absorbance value for each 5′-GMP standard vs. its concentration, from which the 5′-GMP concentration of each sample was determined.

### Detection of PKG activity

Detection of PKG was performed in accordance with the previous literature. Rat aortic rings were rapidly frozen in liquid nitrogen after incubation with saline or different concentrations of NaHS (3, 50 and 300 μmol/L) for 15 min. Thoracic aortic rings were homogenized and extracted in ice-cold protein lysis buffer containing 50 mmol/L Tris-Cl (pH 7.4), 150 mmol/L NaCl, 1 mmol/L ethylenediamine tetra-acetic acid, 1% NP-40, 0.25% sodium deoxycholate, 1 mmol/L phenyl methyl sulphonyl fluoride (PMSF), protease inhibitors, and phosphatase inhibitors. Western blotting analysis was used to detect the phosphorylation level of a PKG substrate (vasodilator-stimulated phosphoprotein, VASP), which reflected PKG activity. The experimental steps were the same as the non-denaturing western blotting. Western blotting analyses were performed according to a previous method, and the primary antibodies were phospho-VASP (Ser-239) antibody (Cell Signaling, 1:1000 dilution) and VASP antibody (Cell Signaling, 1:1000 dilution).

### Primary rat artery smooth muscle cell culture and lentivirus harboring CSE of ASMCs

Rat ASMCs were purchased from Wuhan PriCells Biomedical Technology Co., Ltd (Wuhan, China) and cultured in DMEM (Invitrogen, USA) supplemented with 10% FBS, 100 U/mL penicillin, 2 mmol/L glutamine, 100 μg/mL streptomycin, and primary vascular smooth muscle cell growth supplement (Wuhan PriCells Biomedical Technology Co., Ltd, China). ASMCs were cultured in an incubator at 37°C with 5% CO_2_ and saturated humidity, and the 4th to 6th generations were used for the experiments. ASMCs were seeded in culture flasks of 25 cm^2^ for virus transfection. When cells were grown to 40% to 50% confluence, lentivirus containing the cDNA encoding CSE (Vigene Bioscience, Jinan, China) was added to infect ASMCs for 24 h at a multiplicity of infection of 20. The cells were selected with puromycin (4 μg/mL) to acquire the stably transfected cells expressing CSE.

### Statistical analysis

The results were expressed as means ± SEM. The significant difference between two groups was analyzed by Student's *t* test. Mean values of more than two groups were compared using one-way ANOVA test, with the Student-Newman-Keuls test for post hoc testing of multiple comparisons. *P* values less than 0.05 were considered statistically significant. Half-maximal effective concentration (EC50) for the H_2_S relaxation curve represented the corresponding concentration of H_2_S that achieved 50% of the maximum relaxation effect. SPSS20.0 (SPSS, Chicago, IL) and GraphPad (GraphPad Software, San Diego, CA) software were used for statistical analysis and graphing.

## References

[1] Kimura H (2014). Production and physiological effects of hydrogen sulfide. Antioxid Redox Signal.

[2] Bian JS, Olson KR, Zhu YC (2016). Hydrogen sulfide: biogenesis, physiology, and pathology. Oxid Med Cell Longev.

[3] Wang R (2002). Two’s company, three’s a crowd: can H2S be the third endogenous gaseous transmitter?. FASEB J.

[4] Pearson RJ, Wilson T, Wang R (2006). Endogenous hydrogen sulfide and the cardiovascular system-what’s the smell all about?. Clin Invest Med.

[5] Wang R (2011). Signaling pathways for the vascular effects of hydrogen sulfide. Curr Opin Nephrol Hypertens.

[6] Yang G, Wang R (2015). H2S and blood vessels: An overview. Handb Exp Pharmacol.

[7] Wang R, Szabo C, Ichinose F, Ahmed A, Whiteman M, Papapetropoulos A (2015). The role of H2S bioavailability in endothelial dysfunction. Trends Pharmacol Sci.

[8] Magierowski M, Jasnos K, Kwiecień S, Brzozowski T (2013). Role of hydrogen sulfide in the physiology of gastrointestinal tract and in the mechanism of gastroprotection. Postepy Hig Med Dosw.

[9] Li L, Liu D, Bu D, Chen S, Wu J, Tang C, Du J, Jin H (2013). Brg1-dependent epigenetic control of vascular smooth muscle cell proliferation by hydrogen sulfide. Biochim Biophys Acta.

[10] Holwerda KM, Karumanchi SA, Lely AT (2015). Hydrogen sulfide: role in vascular physiology and pathology. Curr Opin Nephrol Hypertens.

[11] Tang C, Li X, Du J (2006). Hydrogen sulfide as a new endogenous gaseous transmitter in the cardiovascular system. Curr Vasc Pharmacol.

[12] Sun Y, Huang Y, Zhang R, Chen Q, Chen J, Zong Y, Liu J, Feng S, Liu AD, Holmberg L, Liu D, Tang C, Du J (2015). Hydrogen sulfide upregulates KATP channel expression in vascular smooth muscle cells of spontaneously hypertensive rats. J Mol Med (Berl).

[13] Yan H, Du J, Tang C (2004). The possible role of hydrogen sulfide on the pathogenesis of spontaneous hypertension in rats. Biochem Biophys Res Commun.

[14] Li X, Du J, Jin H, Tang X, Bu D, Tang C (2007). The regulatory effect of endogenous hydrogen sulfide on pulmonary vascular structure and gasotransmitters in rats with high pulmonary blood flow. Life Sci.

[15] Yang G, Wu L, Jiang B, Yang W, Qi J, Cao K, Meng Q, Mustafa AK, Mu W, Zhang S, Snyder SH, Wang R (2008). H2S as a physiologic vasorelaxant: hypertension in mice with deletion of cystathionine gamma-lyase. Science.

[16] Gong H, Chen Z, Zhang X, Li Y, Zhang J, Chen Y, Ding Y, Zhang G, Yang C, Zhu Y, Zou Y (2015). Urotensin II protects cardiomyocytes from apoptosis induced by oxidative stress through the CSE/H2S pathway. Int J Mol Sci.

[17] Liu YH, Lu M, Hu LF, Wong PT, Webb GD, Bian JS (2012). Hydrogen sulfide in the mammalian cardiovascular system. Antioxid Redox Signal.

[18] Liu YH, Yan CD, Bian JS (2011). Hydrogen sulfide: a novel signaling molecule in the vascular system. J Cardiovasc Pharmacol.

[19] Kimura H (2014). The physiological role of hydrogen sulfide and beyond. Nitric Oxide.

[20] Kimura H (2012). [Hydrogen sulfide: production, release, and functions]. [Article in Japanese] Nihon Yakurigaku Zasshi.

[21] Zhang J, Chen S, Liu H, Zhang B, Zhao Y, Ma K, Zhao D, Wang Q, Ma H, Zhang Z (2013). Hydrogen sulfide prevents hydrogen peroxide-induced activation of epithelial sodium channel through a PTEN/PI (3,4,5) P3 dependent pathway. PLoS One.

[22] Chen Y, Zhao J, Du J, Xu G, Tang C, Geng B (2012). Hydrogen sulfide regulates cardiac sarcoplasmic reticulum Ca(2+) uptake via K(ATP) channel and PI3K/Akt pathway. Life Sci.

[23] Tang G, Wu L, Wang R (2010). Interaction of hydrogen sulfide with ion channels. Clin Exp Pharmacol Physiol.

[24] Huang J, Luo YL, Hao Y, Zhang YL, Chen PX, Xu JW, Chen MH, Luo YF, Zhong NS, Xu J, Zhou WL (2014). Cellular mechanism underlying hydrogen sulfide induced mouse tracheal smooth muscle relaxation: role of BKCa. Eur J Pharmacol.

[25] Sun Y, Tang CS, Du JB, Jin HF (2011). Hydrogen sulfide and vascular relaxation. Chin Med J (Engl).

[26] Wang R (2011). Signaling pathways for the vascular effects of hydrogen sulfide. Curr Opin Nephrol Hypertens.

[27] Bucci M, Papapetropoulos A, Vellecco V, Zhou Z, Zaid A, Giannogonas P, Cantalupo A, Dhayade S, Karalis KP, Wang R, Feil R, Cirino G (2012). cGMP-dependent protein kinase contributes to hydrogen sulfide-stimulated vasorelaxation. PLoS One.

[28] Liu J, Chen Z, Ye L, Liu H, Dou D, Liu L, Yu X, Gao Y (2014). Preservation of nitric oxide-induced relaxation of porcine coronary artery: roles of the dimers of soluble guanylylcyclase, phosphodiesterase type 5, and cGMP-dependent protein kinase. Pflügers Archiv.

[29] Mazza R, Pasqua T, Cerra MC, Angelone T, Gattuso A (2013). Akt/eNOS signaling and PLN S-sulfhydration are involved in HS-dependent cardiac effects in frog and rat. Am J Physiol Regul Integr Comp Physiol.

[30] Mustafa AK, Gadalla MM, Sen N, Kim S, Mu W, Gazi SK, Barrow RK, Yang G, Wang R, Snyder SH (2009). H2S signals through protein s-sulfhydration. Sci Signal.

[31] Zhao K, Li S, Wu L, Lai C, Yang G (2014). Hydrogen sulfide represses androgen receptor transactivation by targeting at the second zinc finger module. J Biol Chem.

[32] Bibli SI, Yang G, Zhou Z, Wang R, Topouzis S, Papapetropoulos A Role of cGMP in hydrogen sulfide signaling. Nitric Oxide.

[33] Meng G, Wang J, Xiao Y, Bai W, Xie L, Shan L, Moore PK, Ji Y (2015). GYY4137 protects against myocardial ischemia and reperfusion injury by attenuating oxidative stress and apoptosis in rats. J Biomed Res.

[34] Liu Z, Han Y, Li L, Lu H, Meng G, Li X, Shirhan M, Peh MT, Xie L, Zhou S, Wang X, Chen Q, Dai W (2013). The hydrogen sulfide donor, GYY4137, exhibits anti-atherosclerotic activity in high fat fed apolipoprotein E(−/−) mice. Br J Pharmacol.

[35] Elies J, Scragg JL, Huang S, Dallas ML, Huang D, MacDougall D, Boyle JP, Gamper N, Peers C (2014). Hydrogen sulfide inhibits Cav3.2 T-type Ca2+ channels. FASEB J.

[36] Sun Y, Tang CS, Jin HF, Du JB (2011). The vasorelaxing effect of hydrogen sulfide on isolated rat aortic rings versus pulmonary artery rings. Acta Pharmacol Sin.

[37] Avanzato D, Merlino A, Porrera S, Wang R, Munaron L, Mancardi D (2014). Role of calcium channels in the protective effect of hydrogen sulfide in rat cardiomyoblasts. Cell Physiol Biochem.

[38] Bucci M, Mirone V, Di Lorenzo A, Vellecco V, Roviezzo F, Brancaleone V, Ciro I, Cirino G Hydrogen sulphide is involved in testosterone vascular effect. Eur Urol.

[39] Geng B, Cui Y, Zhao J, Yu F, Zhu Y, Xu G, Zhang Z, Tang C, Du J (2007). Hydrogen sulfide downregulates the aortic L-arginine/nitric oxide pathway in rats. Am J Physiol Regul Integr Comp Physiol.

[40] Módis K, Ju Y, Ahmad A, Untereiner AA, Altaany Z, Wu L, Szabo C, Wang R (2016). S-Sulfhydration of ATP synthase by hydrogen sulfide stimulates mitochondrial bioenergetics. Pharmacol Res.

[41] Kim B, Lee K, Chinannai KS, Ham I, Bu Y, Kim H, Choi HY (2015). Endothelium-independent vasorelaxant effect of ligusticum jeholense root and rhizome on rat thoracic aorta. Molecules.

[42] Yao Q, Huang Y, Liu AD, Zhu M, Liu J, Yan H, Zhang Q, Geng B, Gao Y, Du S, Huang P, Tang C, Du J (2016). The vasodilatory effect of sulfur dioxide via SGC/cGMP/PKG pathway in association with sulfhydryl-dependent dimerization. Am J Physiol Regul Integr Comp Physiol.

